# LOWER URINARY TRACT AND BOWEL SYMPTOMS PREDICT QUALITY OF LIFE IN GAY AND BISEXUAL PROSTATE CANCER SURVIVORS

**DOI:** 10.1093/geroni/igz038.1821

**Published:** 2019-11-08

**Authors:** Kristine M Talley, Elizabeth Polter, Benjamin D Capistrant, William G West, Badrinath Konety, B R Simon Rosser

**Affiliations:** 1 University of Minnesota, Minneapolis, Minnesota, United States; 2 Smith College, Northampton, Massachusetts, United States

## Abstract

Little is known about disparities in prostate cancer survivorship experienced by gay and bisexual men (GBM). However, early evidence suggests GBM may experience worse urinary and bowel symptoms than heterosexual men. This cross-sectional Internet-based survey describes the prevalence of lower urinary tract (LUTS) and bowel symptoms and their associations with physical and mental health related quality of life (QOL) in GBM treated for prostate cancer. This study enrolled 193 men who identified as gay or bisexual and had received prostate cancer treatment. The Expanded Prostate Cancer Index Composite instrument measured LUTS and bowel symptoms. The MOS SF-12 measured physical and mental QOL. Participants had a mean age of 63.4 years, were 5.6 years past treatment, and were treated with prostatectomy (52%), radiation (19%), or combined or systemic treatment (29%). The most common symptoms were nocturia (77%), urinary frequency (67%), urinary leakage (59%), bowel urgency (45%), bowel frequency (35%), and watery bowel movements (34%). Mean scores were 81.4±19.2 for urinary function, 74.5±20.7 for urinary bother, 88.9±12.1 for bowel function, 84.5±16.3 for bowel bother, 52.5±8.8 for physical QOL, and 46.0±11.4 for mental QOL. In multivariable models adjusted for age, race, treatment type, and time since diagnosis, urinary bother was associated with worse physical QOL (Adjusted Mean Difference (AMD): 0.11, 95%CI: 0.02-0.21), and bowel bother was associated with worse mental QOL (AMD: 0.23, 95%CI: 0.05-0.42). LUTS and bowel symptoms were common. Symptom bother rather than function predicted QOL. Understanding these disparities will help tailor treatments for this underserved population.

